# Clinical application of mesenchymal stem cells in rheumatic diseases

**DOI:** 10.1186/s13287-021-02635-9

**Published:** 2021-11-09

**Authors:** Yajing Wang, Dan Ma, Zewen Wu, Baoqi Yang, Rong Li, Xingxing Zhao, Helin Yang, Liyun Zhang

**Affiliations:** 1 Third Hospital of Shanxi Medical University, Shanxi Bethune Hospital, Shanxi Academy of Medical Sciences, Tongji Shanxi Hospital, Taiyuan, 030032 China; 2 Shanxi University of Chinese Medicine, Jinzhong, 030619 Shanxi China

**Keywords:** Mesenchymal stem cells, Rheumatic diseases, Clinical application

## Abstract

Mesenchymal stem cells (MSCs) are pluripotent stem cells derived from mesoderm during early development that are characterized by high self-renewal ability and multidirectional differentiation potential. These cells are present various tissues in the human body and can be cultured in vitro. Under specific conditions, MSCs can differentiate into osteoblasts, neuron-like cells, adipocytes and muscle cells and so on, therefore, have a great application value in cell replacement therapy and tissue repair. In recent years, the application of MSCs in rheumatic diseases has received increasing attention. On the one hand, MSCs have the ability to differentiate into bone and cartilage cells; on the other hand, these stem cells are also involved in immune regulation, resulting in the alleviation of inflammation and anti-fibrotic properties and the promotion of vascular repair, thus bringing new hope for the treatment of rheumatic diseases. This article reviews the clinical progress in MSC application for the treatment of rheumatic diseases.

## Introduction

**Fig. 1:**
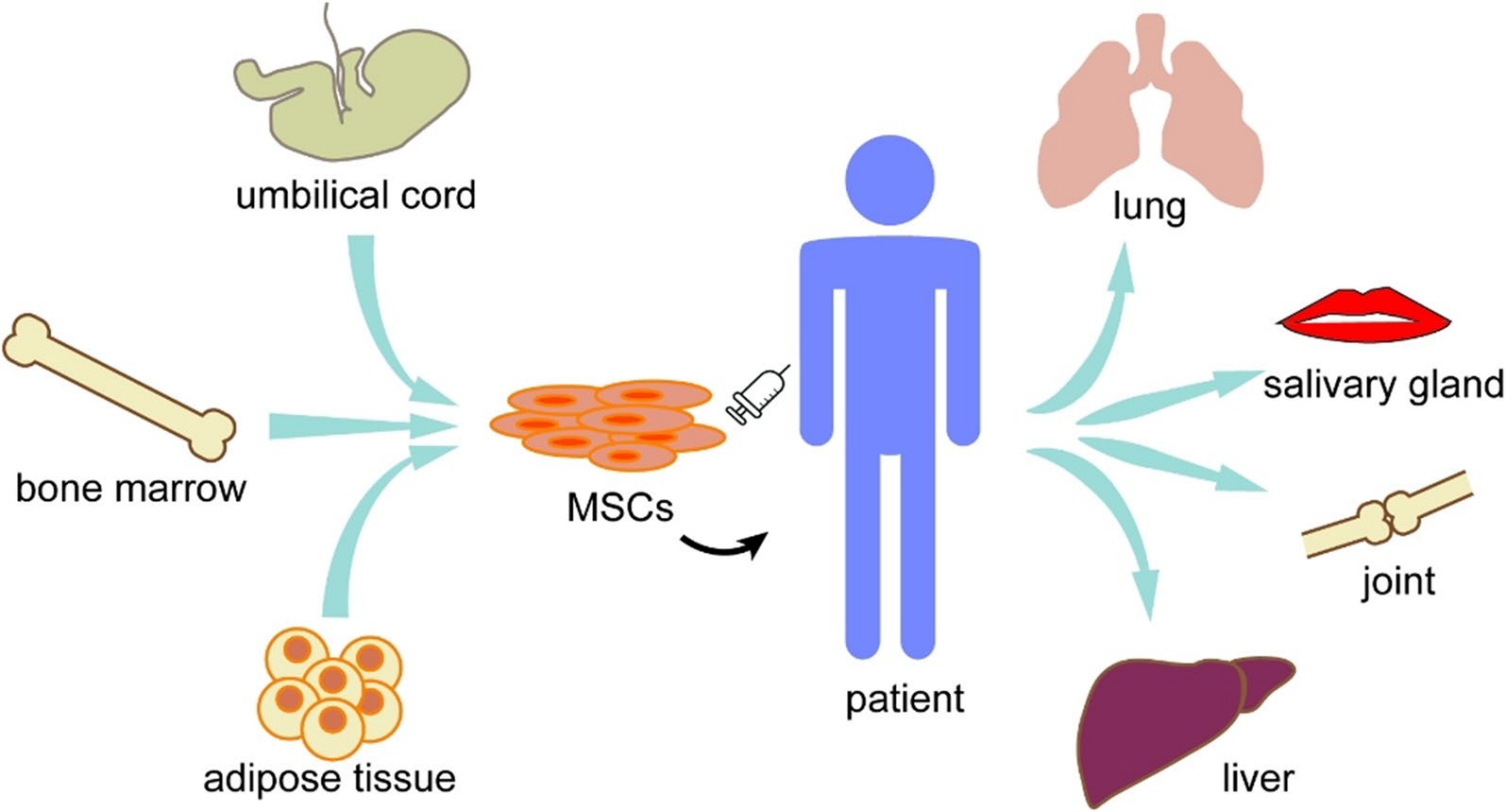
MSCT in rheumatic diseases

Mesenchymal stem cells (MSCs) are derived from the early mesoderm and have a high degree of self-renewal ability and multidirectional differentiation potential [1]. In 1970, Friedenstein et al. discovered that fibroid cells from bone marrow that were cultured and proliferated in vitro could be transplanted under the skin to form bone tissue and rebuild the blood microenvironment [2]. Numerous studies have shown that MSCs can be obtained from a wide variety of sources, including bone marrow (BM), skin, adipose tissue (AD), umbilical cord (UC), and other tissues [3]. Besides their multi-lineage differentiation potential [4–6], MSCs also harbor immunosuppressive activities owing to their paracrine effects and interaction with different immune cells [7–10], and limited immunogenicity with low human leukocyte antigen (HLA) I and no HLA II expression [11]. Studies have shown that BM-MSCs induce low reactivity of T lymphocytes, regulate the expression of inflammatory mediators, significantly reduce the levels of serum tumor necrosis factor α (TNF-α), produce regulatory T (Treg) cells, and prevent severe bone and cartilage damage [12]. The immunomodulatory effects and tissue repair abilities of MSCs indicate their potential for the treatment of serious refractory autoimmune diseases (Fig. 1).

### MSCs and rheumatoid arthritis

Rheumatoid arthritis (RA) is a common systemic autoimmune disease characterized by synovial hyperplasia and joint damage, leading to clinically significant functional impairment and resulting in decreased quality of life. RA patients have an increased risk of atherosclerosis, which leads to cardiovascular problems—a serious threat to human health and life [13]. Significant progress has been made over the last decade to develop targeted therapies for RA. Conventional drugs for RA include anti-inflammatory drugs, corticosteroids, and synthetic disease-modifying antirheumatic drugs (synthetic DMARDs), such as methotrexate (MTX), sulfasalazine, hydroxychloroquine, and leflunomide. In recent years, treatment options for RA have increased, and biological DMARDs, such as anti-TNF-α blocker, anakinra, abatacept, and rituximab, often in combination with MTX, have been shown to be highly effective [14]. However, these therapies are still limited by low efficacy in some patients and serious complications, including infections and malignancies. Therefore, there is a need to identify new treatment options for RA. MSCs have been shown to regulate their local environment, activate endogenous progenitors through cell–cell interactions and the secretion of various factors, and play a role in tissue damage repair. MSCs can also produce a variety of growth factors and cytokines, such as transforming growth factor beta 1 (TGF-β1), vascular endothelial growth factor (VEGF), Interleukin-6 (IL-6), and chemokines (such as MCP-1), which play an important role in tissue repair and remodeling [15, 16]. These characteristics make MSCs an ideal therapeutic agent for treating RA.

Sun et al. [14] reported that intravenous infusions of 1 × 10^6^ MSCs per kg of body weight into four refractory RA patients failed to induce remission; however, no serious adverse events were observed, and authors hypothesized that the relatively low doses of infused MSCs may have been one of the reasons for the lack of success. In the study by Ghoryani et al., autologous BM-MSCs were used to treat nine refractory RA patients [17]. All patients were administered a single intravenous injection of autologous BM-MSCs (1 × 10^6^ cells/kg). After MSCT, 28-joint Disease Activity Score (DAS28), visual analog scale (VAS), and erythrocyte sedimentation rate (ESR) values were significantly reduced, but no adverse events were observed. These findings indicate that autologous BM-MSCs can alleviate the severity and activity of refractory RA.

Wang et al. [18] demonstrated that the intravenous infusion of UC-MSCs (1 × 10^7^ cells per kg of body weight) resulted in positive outcomes in 17 RA patients: all patients had significant improvements in their diet, sleep, physical strength, and fatigue after the infusion, while the routine blood tests of liver and kidney function showed that serum immunoglobulin, C3, and C4 results did not change significantly, and the disease activity decreased.

In 2013, a randomized controlled trial using intravenous infusion of UC-MSCs (4 × 10^7^ cells) in 172 patients with active RA was reported [19]. A total of 136 patients in the experimental group were regrouped and infused twice with UC-MSCs at intervals of 3, 6, and 8 months. The results showed that experimental groups had higher improvement rates compared to the control group. In another multicenter, single-blind, randomized controlled phase Ib/IIa trial [20], 46 patients were randomly divided into three groups based on the intravenous injection of adipose-derived MSCs (1, 2, 4 × 10^6^ cells/kg) on days 1, 5, and 18. A total of 141 adverse events were reported, of which 133 were mild or moderate, and there were no malignant tumors, thromboembolic events, or deaths. The clinical benefits to RA patients who were administered MSCs appeared to diminish or fluctuate after 3 months, suggesting the need for a second transplant.

Park et al. [21] reported another clinical phase Ia study that involved nine patients with refractory RA. These nine patients were divided into three groups and received a single intravenous infusion of 2.5 × 10^7^, 5 × 10^7^, and 1 × 10^8^ UC-MSCs. The results showed that all patients had improved symptoms and serology, and there were no serious adverse events; however, the sample size of this study was very small, the follow-up time was short, and there was no placebo group for comparison.

Gowhari et al. [22] demonstrated that MSCT inhibited B cells by reducing the production of B-cell activating factor (BAFF) and a proliferation-inducing ligand (APRIL) and decreasing the expression of their receptors on the surface of B cells. The plasma levels of BAFF and APRIL declined significantly after MSCT, indicating a significant effect of MSC on body fluid response. These findings suggest that BAFF could be a favorable target for further investigation of RA pathogenesis.

In summary, these results indicate that MSCs can safely and effectively treat RA, significantly improve the clinical symptoms of patients, improve the quality of life, and prevent disease progression; furthermore, second transplantation can further boost the effect of the first transplant. Therefore, MSCs could become a new choice for the clinical treatment of RA.

### MSCs and ankylosing spondylitis

Ankylosing spondylitis (AS) is a chronic progressive type of spinal inflammatory arthritis. Clinical manifestations of AS usually emerge during the third decade of life [23, 24]. AS characteristically affects the sacroiliac joints, axial skeleton, entheses (tendon or ligament attachments to bone), as well as extraskeletal sites, such as the bowel [25], eye [26], and skin [27]. Inflammatory processes associated with AS lead to bone erosion, new bone formation, and ankylosis in the spine, resulting in severe pain, reduction of spinal mobility, and stiffness [23]. TGF-β, Prostaglandin E2 (PGE2), and HLA-G5 are potent immunomodulatory molecules produced by MSCs that suppress the immune system by inhibiting dendritic cell maturation and inducing Treg cell production. Furthermore, MSCs inhibit the proliferation and activity of effector T cells, such as Th1, Th17, and cytotoxic T lymphocytes (CTL), the cells involved in the pathogenesis of AS. In addition, indolamine (IDO) and PGE2 produced by MSCs induce the switch from the pro-inflammatory (M1) to anti-inflammatory (M2) macrophage phenotype [28–31]. Therefore, MSC therapy could be part of cell therapy methods to improve the treatment of AS.

Wang et al. [32] demonstrated that the intravenous infusion of allogeneic BM-MSCs is an effective and safe treatment approach for patients with active AS. In a study by Li et al. [33], the intravenous transfusion of UC-MSCs showed beneficial outcomes, including safety and decreased clinical symptoms in patients with AS. However, additional studies investigating the effectiveness of MSCT and the systemic adverse effects of intravenous injections for the treatment of AS are necessary [32–34], particularly, since recent studies have been conducted on a smaller number of patients due to the low number of AS cases, especially in Asia [32, 33].

### MSCs and systemic lupus erythematosus

Systemic lupus erythematosus (SLE) is a heterogeneous chronic autoimmune disease characterized by the appearance of multiple autoantibodies, including antinuclear antibodies (ANA), and multi-system involvement. It has been shown that genetic susceptibility and external factors, such as drugs, ultraviolet light, infection, and stress, are involved in the pathogenesis of SLE [35]. Regardless of the inducing factors, the mechanism of SLE involves the activation of B cells, production of autoantibodies, and formation of immune complexes [36].

Studies have shown that autologous MSCs do not improve SLE symptoms [37]. However, in 2010, Sun et al. [38] treated 16 active SLE patients with a single intravenous injection of 1 × 10^6^ /kg.

UC-MSCs and demonstrated a significant improvement in serum biological indicators and renal function. Subsequently, Sun et al. [39] performed a phase II clinical study with a single intravenous dose of 1 × 10^6^/kg allogeneic BM- or UC-MSCs in 87 patients with refractory SLE and reported that the complete remission rate at the 4-year follow-up was 50%, while the overall survival rate was 94%. These results indicated that MSCT clinically improved organ dysfunction in patients with refractory SLE and had good clinical safety, since all observed adverse events were not unrelated to the therapy. In another study [40], 40 SLE patients from four clinical centers were transplanted twice with UC-MSCs (1 × 10^6^ cells/kg, injected one week apart). All patients tolerated the treatment well, and no transplant-related adverse events were reported; however, seven patients relapsed 6 months after transplantation, suggesting the need for a second transplantation to prevent recurrence. Similar results were reported by Yang et al. [41], showing that the MSCs dose was positively correlated with efficacy in their study.

To observe possible adverse events and to evaluate long-term safety, nine SLE patients were infused twice with BM-MSCs (1 × 10^6^ cells/kg, one week apart) and followed up for 6 years [42]. No other adverse events, such as palpitation, headache, nausea, or vomiting, were observed. Furthermore, during the 6-year follow-up, the serum tumor markers AFP, CEA, CA125, and CA199 did not increase before and after the transplantation. In another long-term retrospective study, 178 SLE patients were injected intravenously with 1 × 10^6^ cells/kg UC-MSCs [43]. During the follow-up process, 18 patients developed hyperacute adverse events, 15 patients developed herpes zoster, two patients developed cancer, and 14 patients died; however, these events were considered to be unrelated to transplantation.

There is a general consensus that 60% of lupus patients will develop clinically relevant nephritis at some time during the course of their illness, and lupus nephritis (LN) is a major cause of mortality among lupus patients [44]. In an open-label single-center clinical trial, 81 patients with active and refractory LN were administered a single dose of 1 × 10^6^/kg MSCs [45]. The results of this trial demonstrated that the overall survival rate during the 12-month follow-up period was 95% (77/81). LN activity, as assessed using the BILAG score, decreased significantly by the 12-month follow-up. Total disease activity, as assessed using the SLEDAI score, improved significantly after allogeneic MSCT, while no transplantation-related adverse events were observed. These results indicated that allogeneic MSCT resulted in renal remission in active LN patients by the 12-month follow-up, confirming it as a potential therapy for refractory LN.

It was previously reported that both frequency and function of Treg cells are decreased in patients with active SLE [46]; however, both could be restored by corticosteroid treatment that induced disease remission [47], suggesting a role for Treg cells in the pathogenesis of human SLE. It has been shown that the transcription factor FoxP3 acts as a key molecule in Treg development and function and is, therefore, widely used as a nuclear marker for these cells [48]. Sun et al*.* observed that UC-MSCT markedly upregulated the percentage of CD4^+^FoxP3^+^ Treg cells in peripheral blood mononuclear cells three months after transplantation [38]. In addition, the restoration of Treg cells was associated with a concomitant increase in the expression of TGF-β, the cytokine that plays important roles in Treg cell activation and function [49]. The upregulation of Treg cell pathway could be one of the mechanisms underlying the UC-MSCT.

It has also been reported that MSCT combined with immunosuppressive therapy significantly improves the disease status. Furthermore, its efficacy is dose-dependent and repeated injections can delay the progression of the disease and reduce its recurrence; however, to establish the appropriate dosage and multiple infusion intervals, large-scale experiments need to be performed.

### MSCs and Sjogren's syndrome

Sjogren’s syndrome (SS) is a chronic systemic autoimmune disease characterized by lymphocyte infiltration into exocrine glands, such as the lacrimal glands and salivary glands. The clinical manifestations of SS are complex, and the most common symptoms include dry mouth and eyes, which are often accompanied by organ damage [50]. Some SS patients develop malignant lymphoma [51]. Since MSCs possess high proliferation, immune regulation, and multidirectional differentiation capabilities, these cells can inhibit the proliferation and differentiation of various immune cells, secretion of inflammatory factors, and production of antibodies, while promoting the repair of damaged tissues. Therefore, MSC administration has been utilized as a new therapy to treat SS.

In a study by Xu et al., 24 SS patients (with 11 cases of salivary gland damage and 13 cases of multiple organ involvement) were intravenously injected UC-MSCs [52]. The results of that study demonstrated that SS symptoms were significantly reduced by MSCT, the Sjogren's syndrome Disease Activity Index (SSDAI) and VAS were improved, the secretion from salivary glands increased, while no related side effects were observed. This study confirmed that the therapeutic effects of MSCs were due to their immunoregulatory activities, such as regulation of CD4^+^ T cells, promotion of Treg and Th2 development, and inhibition of Th17 and Tfh inflammatory responses. Furthermore, the author also demonstrated that the stromal cell-derived factor-1/C-X-C chemokine receptor type 4 (SDF-1/CXCR4) axis plays an important role in improving the function of salivary glands. Several studies have shown that MSCs inhibit the proliferation of CD4^+^ T cells in non-obese diabetic (NOD) mice [53], inhibit Th17 cell differentiation [54], reduce IL-17A expression, and improve the secretory function of salivary and lacrimal glands in NOD mice. Experimental results have indicated that the defective immune function of BM-MSCs in SS patients may be responsible for the occurrence of SS [52].

In summary, UC-MSCT can significantly increase saliva flow rate, improve clinical symptoms, and inhibit inflammation. Therefore, allogeneic MSCT could be potentially used as a new, effective, and safe treatment method.

### MSCs and polymyositis/dermatomyositis

Polymyositis/dermatomyositis (PM/DM) is an autoimmune disease characterized by weakness of proximal skeletal muscles and obvious skin manifestations, and is known to affect multiple organs, such as muscles, lungs, and kidneys [55]. Currently, the etiology is not known. Several studies have suggested that T helper (Th) cells are involved in the pathogenesis of PM/DM, since Th cell-related cellular dysfunction plays an important role in the occurrence and development of PM or DM [56]. Therefore, MSCT could provide a new therapeutic strategy for the treatment of PM and DM. Several studies have demonstrated that this approach has promising clinical outcomes.

In a long-term retrospective study by Liang et al. [43], 32 PM and DM patients were injected intravenously with 1 × 10^6^/kg MSCs. The results of the 9-year follow-up study demonstrated that the symptoms and serological indicators of patients improved, showing the effectiveness and safety of MSCT in PM and DM, while 11 patients died due to reasons not related to transplantation.

In another study by Lai et al., 81 patients with PM/DM were randomly divided into two groups: 44 patients in the control group were individually treated with glucocorticoids and immunosuppressants for 6 months, while 37 patients in the transplantation group were injected intravenously with 3.5–5.2 × 10^7^ UC-MSCs [56]. The results of that study showed that the creatine kinase values in both groups were significantly decreased; however, the transplantation group had better results than the control group at several time points, and the lung function was significantly improved in the transplantation group. One patient died after transplantation and no transplantation-related complications occurred. Wang et al. [57] reported similar results, showing the efficacy and safety of MSCT for the treatment of PM/DM. Therefore, glucocorticoid and immunosuppressive therapy combined with UC-MSCT is a safe and effective option to treat PM or DM.

Lai et al. observed that after UC-MSCT, the levels of IFN-γ increased, while those of interleukin 4 significantly decreased. It was proposed that Th1 and related secreted pro-inflammatory factors were downregulated in response to UC-MSCT. The increase in IL-4 levels indicated the upregulation of Th2 cells, suggesting that UC-MSCs could be involved in the regulation of Th cell balance, resulting in the alleviation of clinical symptoms in patients with PM/DM [56].

Currently, there are only a few studies investigating PM/DM, and large-scale and randomized clinical studies are needed to evaluate the long-term effectiveness and safety of MSCT in PM/DM patients, including the risks of tumors and infections, as well as the optimal transplantation dose and schedule (Table 1).

**Table 1:** Clinical application of mesenchymal stem cells in rheumatic diseases

Disease	Source	Doses	Cases	Styles	Effectiveness	Safety	References
RA	1BM 3UC	1 × 10^6^/ kg	4	i.v	3 Relapse 1 Invalided	Safe	[14]
	UC	1 × 10^7^cells	17	i.v	Modify	Safe	[17]
	UC	4 × 10^7^cells × 2 (M0.3)	76	i.v	Modify	Safe	[18]
		4 × 10^7^cells × 2 (M0.6)	45				
		4 × 10^7^cells × 2 (M0.8)	15				
	UC	2.5 × 10^7^cells	3	i.v	Modify	Safe	[19]
		5 × 10^7^cells	3				
		1 × 10^8^cells	3				
	AD	1 × 10^6^/ kg × 3 (D1.5.18)	20	i.v	Modify	Safe	[20]
		2 × 10^6^/ kg × 3 (D1.5.18)	20				
		4 × 10^6^/ kg × 3 (D1.5.18)	6				
	BM (Autologous)	1 × 10^6^/ kg	9	i.v	Modify	Safe	[21]
AS	BM	1 × 10^6^/ kg (D0.7.14.21)	4	i.v	Modify	Safe	[32]
	UC	8 × 10^7^cells (M0) 7 × 10^7^cells (M3)	5	i.v	Modify	Safe	[33]
SLE	UC	1 × 10^6^/ kg	16	i.v	Modify	Safe	[38]
	BM	1 × 10^6^/ kg	26	i.v	20 Relapse	4 Infection 5 Died	[39]
	UC	1 × 10^6^/ kg	61	i.v			
	UC	3 × 10^7^cells	20	i.v	8 Relapse	Safe	[40]
		3 × 10^7^cells × 2 (D0.7)	20				
	UC	1 × 10^6^/kg × 2 (D0.7)	40	i.v	7 Relapse	Safe	[41]
	UC	1 × 10^6^/kg × 2 (D0.7)	9	i.v	Modify	1 Died 14 Infection	[42]
	UC	1 × 10^6^/kg	178	i.v	Modify	14 Died	[43]
	23BM 58UC	1 × 10^6^/kg	81	i.v	Modify	4 Died 4 Infection	[45]
pSS	UC	1 × 10^6^/kg	24	i.v	Modify	Safe	[52]
PM/DM	UC	1 × 10^6^/kg	30	i.v	Modify	11 Died	[43]
	UC	3.5–5.2 × 10^7^cells	37	i.v	Modify	1 Died	[56]
	UC/BM	1 × 10^6^/kg	10	i.v	Modify	Safe	[57]
SSc	UC	1 × 10^6^/kg	39	i.v	Modify	6 Died	[43]
	BM	0.2–1.8 × 10^6^/kg	5	i.v	Modify	1 Died	[62]
	BM	1 × 10^6^/kg	14	i.v	Modify	5 Infection	[64]

### MSCs and systemic sclerosis

Systemic sclerosis (SSc) is a chronic autoimmune disease characterized by increased synthesis and deposition of extra-cellular matrix in skin and various internal organs. The abnormal deposition of collagen in the skin and other organs leads to multiple organ dysfunction, and the prognosis of individuals with lung, heart, or kidney involvement is poor [58]. Fibrosis is not reversible, and there is no safe and effective treatment for SSc [59]. MSCs have the potential to differentiate into bone or muscle cells, as well as into endothelial cells. Compared with healthy controls, MSCs from SSc patients exhibit abnormal functional activities, such as increased expression of TGF-βand vascular endothelial growth factor (VEGF), and impairment of endothelial cell differentiation, which may play critical roles during the development of fibrosis in SSc [60, 61]. Based on these findings, allogeneic MSCT appears a promising therapy for SSc.

In a long-term retrospective study by Liang et al. [43], 39 patients with SSc were injected intravenously with 1 × 10^6^/kg MSCs. The 9-year follow-up results showed that the skin symptoms and serological indicators of all patients improved, demonstrating the effectiveness and safety of MSCT in the treatment of SSc, while the death of six patients was not related to transplantation.

In a 2011 study by Keyszer et al. [62], five patients with severe SSc were administered the intravenous injections of 0.2–1.8 × 10^6^/kg MSCs. Out of these five patients, three patients had lung involvement (one patient had pulmonary fibrosis), four patients had skin ulcers, and two patients had myositis. Within 6 months, the skin symptoms of four patients improved and limb necrosis was significantly alleviated in three patients. Due to the heterogeneity of the cases, no definite conclusions could be drawn regarding the efficacy of BM-MSCT. However, based on these results, BM-MSCT appears to be safe for SSc patients, and the healing of skin ulcers was the most significant therapeutic effect of this therapy.

In another study, two patients received plasma exchange and rituximab treatment [63], as well as allogeneic UC-MSCs as part of the comprehensive treatment of patients with scleroderma. In an open study [64], 14 patients with SSc were treated with a single injection of BM-MSCs combined with plasma exchange. The results of this study showed that the MRSS decreased, while the anti-Scl70 antibody titer increased, and lung parameters were also improved.

### Conclusions and outlook

Bone marrow and umbilical cord are convenient sources of stem cells, and MSCs can be easily derived from these tissues. For MSCT, intravenous injections are commonly used and the dosage generally varies between 1 × 10^6^ cells/kg and 1 × 10^8^ cells/kg. Based on the studies described in this review, the repeated injections of MSCT appear to be more effective than a single administration. Furthermore, the effectiveness of MSCT in rheumatic diseases has been widely confirmed. In terms of safety, infections and cancer are two important and significant issues related to MSCT. Long-term follow-up studies showed that serum tumor markers did not increase before and 6 years after MSCT [42]; however, there are no reports investigating the correlation between MSCT and the incidence of infection and cancer. The regulatory mechanism of MSCT for different autoimmune diseases has not yet been fully elucidated, and there is still a lack of large-scale and randomized multicenter clinical studies on MSCT. Clinical treatment with MSCs requires that the safety and effectiveness of pharmaceutical preparations, as well as the quality and scientific validity of relevant clinical research and trial programs, are first guaranteed. The field of stem cell therapy expects high-quality clinical evidence to support the application of stem cell transplantation. Clinical research can also identify future development directions for basic stem cell research [65]. However, as a cell therapy, MSCT may cause capillary bed blockage, ectopic osteogenesis, chondrogenesis, tumor and other risks, which limits its clinical application to a certain extent. It is believed that with the gradual improvement of our understanding of the biological properties and clinical applications of MSCs, MSCT will have a wider application. Further research on MSCT will provide new perspectives and ideas for its use in the treatment of autoimmune diseases.

## Data Availability

Please contact the corresponding author for data requests.

## Abbreviations

MSCs: Mesenchymal stem cells
MSCT: Mesenchymal stem cells transplantation
SM: Synovial membrane
BM: Bone marrow
AD: Adipose tissue
UC: Umbilical cord
SM-MSCs: Synovial membrane mesenchymal stem cells
BM-MSCs: Bone marrow mesenchymal stem/stromal cells
RA: Rheumatoid arthritis
BAFF: B-cell activating factor
VEGF: Vascular endothelial growth factor
AS: Ankylosing spondylitis
SLE: Systemic lupus erythematosus
SLEDAI: Systemic Lupus Erythematosus Disease Activity Index
BILAG: British Isles Lupus Assessment Group
SSc: Systemic sclerosis
SS: Sjogren's syndrome
SDF-1/CXCR4: Stromal cell-derived factor-1/C-X-C chemokine receptor type 4
PM: Polymyositis
DM: Dermatomyositis
